# Cambial Growth Season of Brevi-Deciduous *Brachystegia spiciformis* Trees from South Central Africa Restricted to Less than Four Months

**DOI:** 10.1371/journal.pone.0047364

**Published:** 2012-10-10

**Authors:** Valérie Trouet, Mukufute Mukelabai, Anouk Verheyden, Hans Beeckman

**Affiliations:** 1 Laboratory for Tree-Ring Research, University of Arizona, Tucson, Arizona, United States of America; 2 Zambia Meteorological Service, Mongu, Zambia; 3 Geology Department, Union College, Schenectady, New York, United States of America; 4 Laboratory for Wood Biology and Xylarium, Royal Museum for Central Africa, Tervuren, Belgium; DOE Pacific Northwest National Laboratory, United States of America

## Abstract

We investigate cambial growth periodicity in *Brachystegia spiciformis*, a dominant tree species in the seasonally dry miombo woodland of southern Africa. To better understand how the brevi-deciduous (experiencing a short, drought-induced leaf fall period) leaf phenology of this species can be linked to a distinct period of cambial activity, we applied a bi-weekly pinning to six trees in western Zambia over the course of one year. Our results show that the onset and end of cambial growth was synchronous between trees, but was not concurrent with the onset and end of the rainy season. The relatively short (three to four months maximum) cambial growth season corresponded to the core of the rainy season, when 75% of the annual precipitation fell, and to the period when the trees were at full photosynthetic capacity. Tree-ring studies of this species have found a significant relationship between annual tree growth and precipitation, but we did not observe such a correlation at intra-annual resolution in this study. Furthermore, a substantial rainfall event occurring after the end of the cambial growth season did not induce xylem initiation or false ring formation. Low sample replication should be taken into account when interpreting the results of this study, but our findings can be used to refine the carbon allocation component of process-based terrestrial ecosystem models and can thus contribute to a more detailed estimation of the role of the miombo woodland in the terrestrial carbon cycle. Furthermore, we provide a physiological foundation for the use of *Brachystegia spiciformis* tree-ring records in paleoclimate research.

## Introduction

The periodicity of tree growth is controlled by an interaction of genetic and environmental factors [1] and is expressed on intra-annual (seasonal) as well as inter-annual and longer time-scales. In temperate regions, temperature variability is the main climatic driver of tree growth and its seasonality is imprinted in tree growth periodicity. Temperate phenological cycles, including the timing and the length of the growth season, are closely related to environmental factors (temperature, photoperiod) and respond to inter-annual temperature variations [2], as well as to 20^th^ Century warming [3], [4]. Also the development of secondary tree growth is regulated by photoperiod [5] and temperature [6], [7], [8], with threshold temperatures controlling the length of the cambial growth season [7], [9] and seasonal mean temperatures determining the onset of wood formation [10] and shoot growth [11] in spring.

The seasonally-induced growth stop in temperate trees results in the formation of annual tree rings [12], which are used in a plethora of dendrochronological applications, including climate reconstruction, archaeology, forestry, and ecology. In tropical regions, seasonal variations in temperature and photoperiod are limited, and it has long been assumed that tropical trees do not experience a cambial dormancy period and do not form annual rings (see [13], [14] for an overview). The recent development of cross-dated tree-ring chronologies from tropical forests spanning a range of precipitation regimes (e.g., [15], [16], [17]) has since refuted this assumption and suggests that other internal and external factors dominate tree growth periodicity. In seasonally dry tropical forests (SDTFs), tree water status (TWS), a function of both endogenous and seasonal environmental factors, is the primary driver of leaf phenology and cambial activity [18], [19], [20]. SDTFs consist of a mosaic of four main leaf phenological functional types (evergreen, brevi-deciduous, deciduous, and stem succulent trees) which are adapted to drought and respond to seasonal variations in TWS in different ways [18], [20]. Because of the complex mixture of leaf phenological patterns in SDTFs, community-level leaf phenological studies [21], [22], [23] mask diversity and do not reflect the full leaf phenological spectrum. Detailed studies of water relations in the different leaf phenological functional types are therefore essential for understanding the relationships between tree growth and climatic variability in SDTFs [20], [24]. The seasonality of cambial activity in SDTFs and its relation to climatic seasonality has primarily been studied using band and electric point dendrometer measurements [18], [25], [26], [27], [28]. This high (daily) resolution method is non-destructive and low-maintenance and is thus applicable in remote areas, but does not allow distinguishing between stem rehydration phases and phases of actual cambial stem growth [29], [30]. A comparative study of wood formation monitoring methodologies [31] has revealed that pinning and microcoring techniques are more reliable techniques than dendrometer measurements.

Here, we study tree growth periodicity in the miombo woodland, the principal SDTF type of sub-Saharan Africa, covering over 2.7 million square kilometer [32]. Miombo woodland is dominated by three closely related genera from the *Leguminosae* family (*Brachystegia*, *Isoberlinia*, and *Julbernardia*). All three tree species are brevi-deciduous and experience a short period (a few days to several weeks) of drought-induced deciduousness, after which TWS increases and leaf-flushing occurs before the onset of the wet season [18], [20]. We focus our study on *Brachystegia spiciformis* Benth. (*Brsp*), which sheds its leaves during the dry season from July to September [33]. *Brsp* forms annual rings [34], [35], [36], marked by boundaries consisting of axial parenchyma bands. Despite the distinct character of growth ring boundaries, growth ring anomalies (particularly partially absent rings) occur frequently [35]. The influence of precipitation on secondary tree growth, as measured by annual ring widths, is strongest during the core of the rainy season (December-February), when precipitation rates are highest [34], [35].

To better understand how the brevi-deciduous phenology of *Brsp* can be linked to a distinct period of cambial dormancy resulting in the formation of annual growth rings, we here study its cambial activity over one year by applying the pinning method [37]. It is worth noting that complementary analysis methods such as cambial monitoring through the extraction of micro-cores (e.g., [6]) or electron microscopy [38] are necessary for a more detailed analysis of cambial activity, including cell division, cell expansion, and lignification.

Our specific research questions include:

1. At what time of year does cambial activity in *Brsp* start/end and how long is its cambial growth season?
2. Is this growth season synchronous between individual trees?
3. Is there a relation between cambial activity and climatic and environmental factors (precipitation, temperature, relative humidity, photoperiod) throughout the growth season?
4. How does the length and period of the cambial growth season relate to the leaf phenological growth season?

## Methods

### Study site

**Figure 1:**
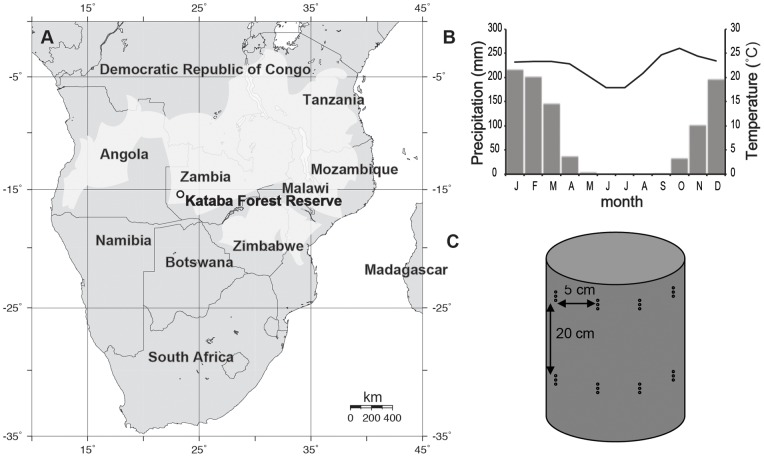
Study site location, climate, and pinning strategy. Location of Kataba Forest Reserve in Zambia (a) and miombo woodland distribution (light grey shading); climate diagram for Mongu meteorological station (b); schematic diagram of pinning application on an individual stem (c). In b, monthly precipitation sums are depicted by grey bars and average monthly temperatures by the black line.

We collected samples at the Kataba Forest Reserve, approximately 20 km South of Mongu in western Zambia (15°26′ S, 23°15′ E; Fig. 1a), on a flat area adjacent to the Zambezi River flood plain. Sampling permits were obtained through the Safari 2000 Southern African Regional Science Initiative. The vegetation at the site consists of miombo woodland, but its structure and species composition are influenced by specific edaphic conditions with generally poor soils derived from deep (30 m) Kalahari sands. The forest canopy at Kataba Forest Reserve has an average height of 12 m and a total tree canopy cover of 49%, measured with a spherical densitometer at 42 circular plots within the forest. Woody plant basal area measured at breast height averages at 8.2 m^2^ ha^−1^ [39]. The forest is dominated by *Brsp*, which occurred in 95% of the 42 plots and was the primary contributor (52%) to the tree canopy cover [40].

The site experiences a seasonally dry tropical climate (Fig. 1b), with a mean annual rainfall (at Mongu meteorological station) of 947 mm yr^−1^ (standard deviation (stdev) = 189 mm; 1905–2005), a mean annual temperature of 22.4°C (stdev  = 0.8°C; 1924–2003), and mean annual relative humidity of 49% (stdev  = 3.3%; 1948–2000). The majority of precipitation falls during the austral summer season, with a dry season (<60 mm month^−1^) lasting seven months from April until October. Three seasons based on temperature and rainfall are prevalent in the miombo region: hot-dry (September-October), hot wet (November-April), and cool dry (May – August) [41] and the leaf phenology of the main miombo species is coordinated with this seasonality [42], [43].

### Sampling strategy

We applied a pinning method [37], [44], [45] to six *Brsp* trees in the Kataba Forest Reserve. This method employs pins or nails, inserted through the bark and cambium into the xylem, to induce a cambial response: formation of new cells is stopped in the immediate vicinity of the pinning canal, whereas modified cells are produced further away from the canal [44]. This distinct reaction to the cambial marking enables detection of the actual phase of intra-annual wood formation at the time of pinning and thus cambial growth analysis.

**Figure 2:**
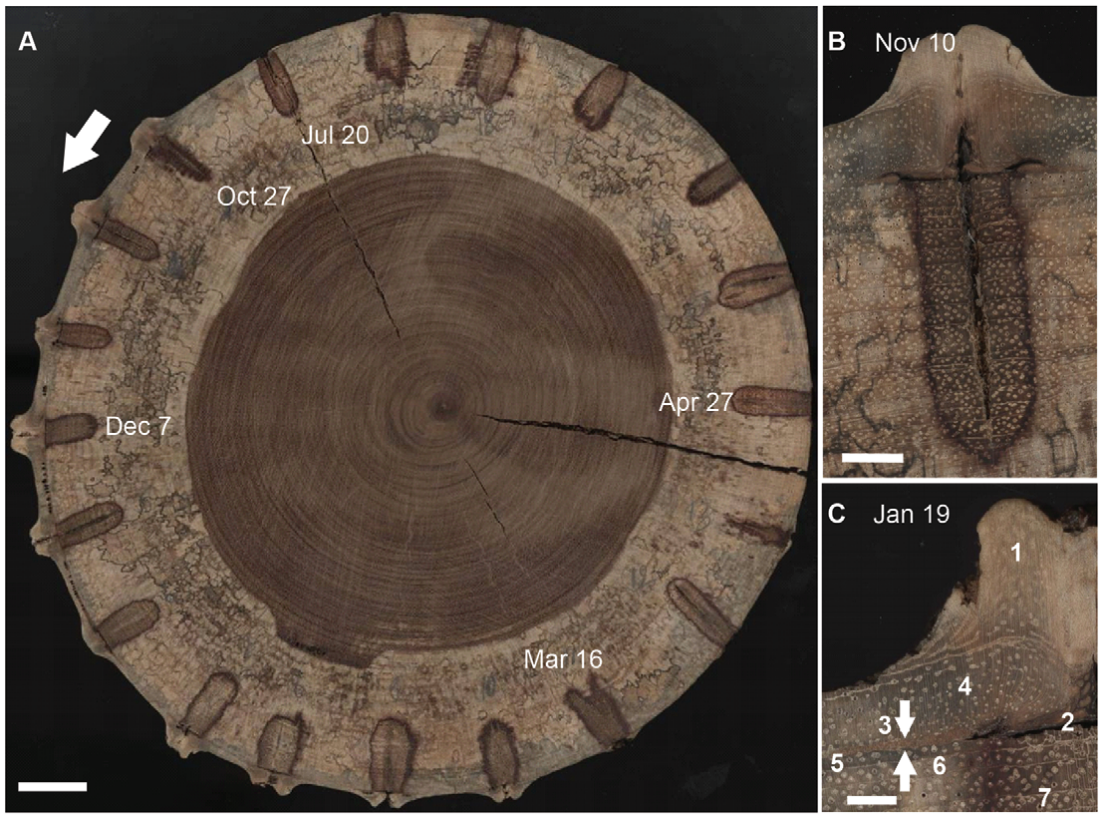
Reaction to bi-weekly pinning in *Brachystegia spiciformis*. (a) Cross-section of a *Brachystegia spiciformis* tree that was pinned on a bi-weekly base between October (Oct) 27, 2001 and July (Jul) 20, 2002. The darker coloured compartmentalization zones around each inserted pin are clearly visible as well as the darker heartwood area in the center of the trunk, compared to the lighter coloured sapwood. Note that pinning continued after Jul 20,2002 (until Oct 17, 2002), but was conducted along a different cross-section of the same tree (see also Fig. 1c). (b) Close-up of the wound formed by the pinning on November (Nov) 10, 2001 and the darker coloured compartmentalization zone that resulted from it. (c) Detail of the wood zone surrounding the pinning on January (Jan) 19, 2002: 1–callus tissue formed as wound reaction to the pinning; 2–wound inflicted by the pinning, including the dark, died-off cambium layer; 3–parenchyma cells (between white arrows) formed as reaction to the pinning; 4–xylem formed after the pinning on Jan 19, 2002 but before the end of the growth season 2001–2002; 5–xylem formed between the start of the growth season 2001–2002 and the pinning on Jan 19, 2002; 6–xylem (growth ring) formed during the growth season 2000–2001; 7–compartmentalization zone. White scale-bar  = 25 mm (a), 5 mm (b), and 2 mm (c).

Over the course of almost one year (October 27, 2001 until October 17, 2002) and on a bi-weekly basis, three nails (1 mm diameter) were hammered into each tree (Fig. 1c) at breast height (1.3 m). By inserting three nails in a vertical line for each pinning, we assured that the markings left by the nails were visible on a cross-section of the stem. Nails were inserted through the bark and cambium into the xylem. All pinned trees were felled in November 2002. We decided to limit our cambial wounding experiment to six trees because of the destructive character of the applied pinning method. Trees were selected to be of comparable diameter (Table 1), to have a straight bole, to be in healthy condition, and to represent the natural vegetation at Kataba Forest Reserve. Stems were cut transversely at pinning height (Fig. 2a) and the stem discs were sanded progressively (grit 100–1200) and polished. All samples are lodged in the Xylarium of the Royal Museum for Central Africa in Tervuren, Belgium (accessions Tw56863 to Tw56871). Tree ring counts (for age determination) and analysis of the wound reaction and of wood formation (including cell counts) after the cambial marking were conducted directly on the polished wood under a microscope with incident light and high magnification. Ring counts revealed that the sampled trees ranged in age between 38 and 50 years at the time of cutting (Table 1). Cell counts were conducted independently by three researchers along 5 to 9 radii close to each pinning wound and the median values of these 5 to 9 cell counts were then used in the analysis.

**Table 1:** Sample-specific tree growth characteristics.

Sample	DBH (cm)	Age (years)	Average TRW (mm)	2001–2002 TRW (mm)	Growth start	Growth end	Growth season length (days)
Tw56863	21	49	216	61	Dec 22	Mar 16	97
Tw56864	25.5	50	256	309	Dec 7	Mar 2	97
Tw56865	18	42	217	269	Dec 7	Mar 16	111
Tw56867	13.5	47	144	73	Dec 22	Mar 2	83
Tw56869	17	38	223	63	Dec 7	Mar 2	97
Tw56871	18	50	181	145	Dec 7	Mar 16	111

Diameter at breast height (DBH), age, average tree-ring width (TRW), TRW in 2001–2002, and start and end date and maximum length of the cambial growth season for 6 *Brsp* trees in Kataba Forest Reserve.

### Instrumental data

The Kataba Forest Reserve functioned as an EOS validation core site in the framework of the Safari 2000 project [46], [47]. Climatic variables (precipitation, relative humidity, and minimum, maximum, and average temperature) were measured on a daily basis for the period from October 2001 until November 2002.

We used the MODIS (Terra satellite, MOD15, level 4, collection 3) leaf area index (LAI) product, which is produced at 1km spatial resolution and over 8-day compositing periods [48], to track LAI seasonality in the Kataba Forest Reserve over the years 2001–2002. For this purpose, LAI values for a window of 7*7 gridpoints, centered on the location of the Kataba Forest Reserve, were averaged. An algorithm based on the inversion of a three-dimensional radiative transfer model is used to produce the MODIS LAI product and it corresponds well to ground-based LAI measurements [49].

Effective LAI measurements, obtained using a LICOR LAI-2000 Plant Canopy Analyzer instrument [50] were measured at 93 locations within the Kataba Forest Reserve and the 93 values were averaged to validate MODIS LAI estimates [51]. Effective LAI measurements were collected throughout the growing season, but measurements were rejected when they were collected under partly cloudy conditions, when open-sky measurements were not available, or when the sky became too dark for meaningful transmittance values to be determined [49], resulting in viable measurements for only four dates during the period October 2001-April 2002.

### Data analysis

Cell counts were conducted independently by three researchers along 5 to 9 radii close to each pinning wound and the median values of these 5 to 9 cell counts were calculated for each tree. To account for inter-tree differences, biweekly cell counts per tree were calculated as percentages of total cell counts at the end of the cambial growth season (cell count at final pinning  = 100%). Biweekly percentages were then averaged for the six trees and the average percentages for subsequent pinnings were differenced to obtain biweekly cambial growth. Bi-weekly percentages of cambial growth were then compared to bi-weekly relative humidity, temperature, and MODIS LAI averages and precipitation sums in a Pearson correlation analysis (using R software v.2.11.0) over the course of the cambial growth season (December 7-March 16, n = 8 biweekly data points).

## Results

### Wound reaction

Throughout the course of the year, pinning provoked distinct wound reactions, which were characterized by a combination of several wood anatomical features (Fig. 2c). A discoloured reaction zone was formed around the pinning canals (Fig. 2a, b): xylem tissue immediately surrounding the canals was stained and demarcated by a thin, darker line corresponding to the position of the wound periderm or compartmentalization zone barrier [52]. This compartmentalization zone, extending up to 15 mm tangentially and up to 3 mm radially from the pinning canal and occurring throughout the entire year, might restrict the inward spread of pathogens that penetrate the wound [53].

**Figure 3:**
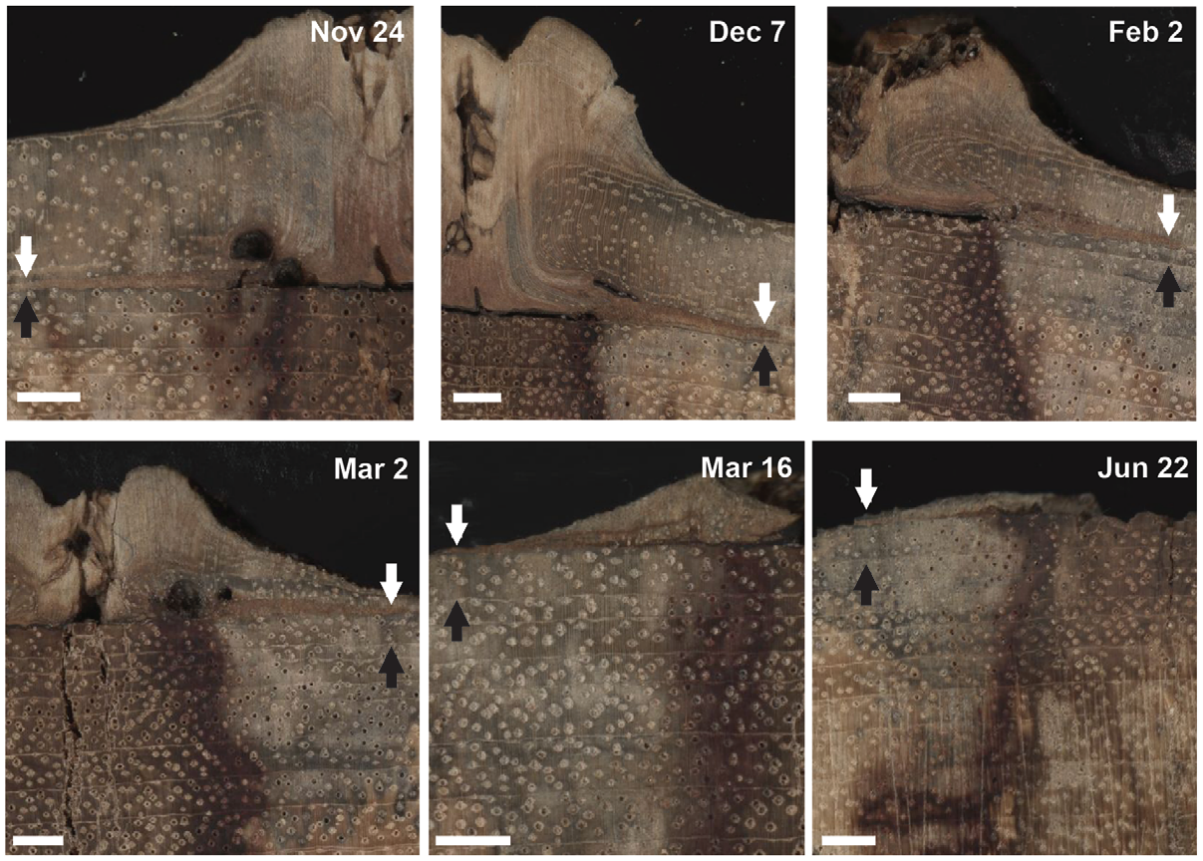
Chronosequence of pinning reactions in *Brachystegia spiciformis* throughout the growth season. Chronosequence of pinning reactions in *Brachystegia spiciformis* between November (Nov) 24, 2001 and June (Jun) 22, 2002. White arrows indicate the parenchyma layer formed as a reaction to the pinning, black arrows indicate the marginal parenchyma band that forms the boundary of the 2000–2001 growth ring. On Nov 24, 2001, the wound parenchyma layer coincides with the 2000–2001 growth ring boundary, whereas on December (Dec) 7, 2001, a small number of xylem (fiber and vessel) cells is visible between the two parenchyma layers, indicating that the 2001–2002 growth season started between Nov 24 and Dec 7, 2001. The number of cells between the two parenchyma layers increases between Dec 7, 2001 and March (Mar) 2, 2002 as the tree continues its woody growth. No regular xylem cells are formed after the pinning on Mar 16, 2002, the wound parenchyma layer corresponds to the outside of the sample at this point in time, indicating the end of the 2001–2002 growth season. Note that callus tissue, as a reaction to the pinning, continues to be formed even after the end of the growth season and throughout the dry season (e.g., Jun 22). White scale-bars  = 2 mm in all photographs.

As a response to the wound caused by pinning, the cambium stopped producing new xylem cells in the immediate vicinity of the wound (2 in Fig. 2c), but produced a layer of anomalous wound parenchyma cells that extended up to 8mm tangentially from the pinning canal (3 in Fig. 2c). In some locations, gum canals were formed as part of the reaction to the wound (e.g., November 24 and March 2 in Fig. 3). A large amount of wound callus tissue (1 in Fig. 2c) was formed by the cambium during the growth season and this amount diminished substantially after the end of the cambial growth season (e.g., March 2 vs. March 16 in Fig. 3). Nevertheless, the cambium continued to produce callus tissue as a reaction to the wounding throughout the year, even long after it stopped producing regular xylem cells at the end of the cambial growth season (e.g., June 22 in Fig. 3).

### Intra-annual growth periodicity

**Figure 4:**
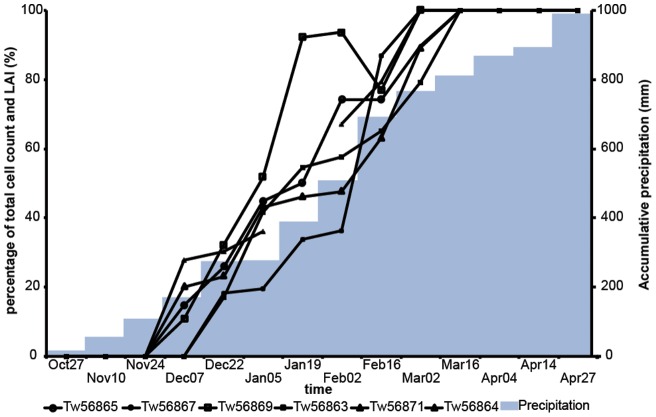
Cumulative xylem formation, and precipitation throughout the growth season. Median percentage of maximum cell counts in 6 *Brachystegia spiciformis* trees (thin solid lines) and cumulative precipitation (gray bars) at Kataba Forest Reserve at bi-weekly intervals between October 27, 2001 and April 27, 2002. The apparently negative growth recorded in Tw56869 on February 16 can be explained by variation in the strength of cambial activity around the circumference of the tree.

We followed intra-annual growth in *Brsp* over the course of one year (October 2001-October 2002). The November 24 pinning, approximately a month after the start of the rainy season, shows that none of the trees had started producing new xylem cells at this point in time (Fig. 4). By December 7, four of the six trees had started their cambial growth (e.g., sample Tw85764 in Fig. 3) and by December 22, this was the case for all trees (Table 1). The pinning experiment thus reveals that the start of the cambial growth season occurs in the month between November 24 and December 22 for all *Brsp* trees in Kataba Forest Reserve. Similar results are found for the end of the cambial growth season: half of the sampled trees had stopped producing new xylem cells by March 2 and the other half by March 16 (Fig. 3 and Fig. 4). For all trees, the end of the cambial growth season thus occurred in the month between February 16 and March 16. It is worth noting that the temporal resolution of our pinning experiment does not allow us to estimate start and end dates at higher than bi-weekly resolution and that the actual difference in cambial growth start or end recorded by two trees on two different pinning dates, could be as short as two days or as long as one month. The cambial growth season for *Brsp* in western Zambia thus has an absolute minimum duration of two (December 22 until February 16) and an absolute maximum of four months (November 24 until March 16).

### Climatic influence on cambial growth

At the start of the cambial growth season, 11% (November 24) to 28% (December 22) of the annual precipitation had fallen (Fig. 4). The cambial growth season ended when 75% (February 16) to 82% (March 16) of the total annual precipitation had been reached. A large individual rainfall event (87 mm in one day) occurred on April 15 after the end of the cambial growth season and did not induce the formation of a second or a false growth ring in any of the trees.

When comparing median bi-weekly cell counts with bi-weekly precipitation sums, temperatures, and relative humidity values over the course of the cambial growth season (December 7 to March 16; n = 8), no significant correlations were found (r = −0.42 for minimum temperature to r = 0.31 for average temperature; p>0.1).

**Figure 5:**
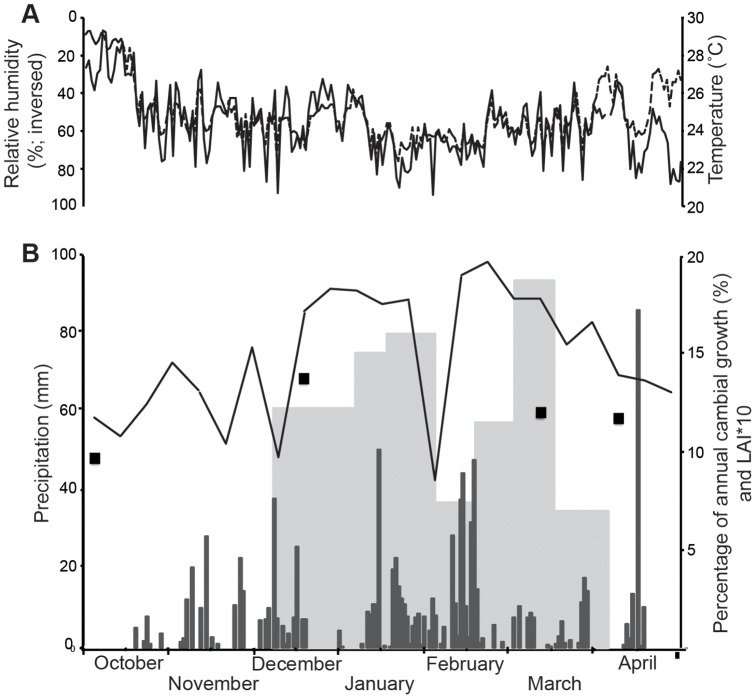
2001–2002 Climate, cambial growth, and LAI at Kataba Forest Reserve. Daily temperature and relative humidity (inversed) (a) and precipitation (b) at Kataba Forest Reserve for the period October 2001-April 2002. Biweekly average percentage of annual *Brachystegia spiciformis* cambial growth and weekly MODIS LAI estimations as well as four LAI 2000 measurements are also indicated in (b).

A comparison of the evolution of the cambial growth season with daily weather conditions (Fig. 5) reveals no clear meteorological trigger for the start and the end of the cambial growth season. There is a remarkable shift in weather conditions at the start of the rainy season in early October, with an approximate 3°C decrease in average daily temperature (27.5°C for Oct 1–10 to 24.3°C for Oct 20–29) and more than a doubling of the relative humidity (from 28% for Oct 1–10 to 68% for Oct 20–29), but this shift occurs almost two months before the start of the cambial growth season and does not appear to influence it.

The October decrease in average daily temperature is primarily caused by a decrease in daily maximum temperatures as a result of an increasing occurrence of clouds and rainfall events with the onset of the rainy season, but no large shifts in maximum or minimum daily temperatures occur over the course of the cambial growth season (results not shown). Also the end of the cambial growth season in late February-March does not appear to be related to any strong shift in weather conditions. In terms of photoperiod, the cambial growth season starts shortly before the start of austral summer when maximum day length is reached: on November 24, day length is at 93% of the annual maximum, which is reached on December 21. The end of the cambial growth season corresponds to day lengths at 74% (February 17) to 53% (March 16) of the annual maximum.

### Leaf phenology

The annual course of LAI as measured by MODIS for the Kataba Forest Reserve shows that maximum LAI (LAI = 2) is reached in late February, after which LAI steadily decreases and reaches a minimum (LAI = 0.9) in Mid-August [49]. Green-up (when many trees begin foliage production, resulting in increasing LAI values) at the forest site starts in early September. It is worth noting that anomalously low LAI values were recorded on November 24, December 10, and February 10 due to cloud cover (Fig. 5) and were removed from the time-series for further analysis. LAI levels during the cambial growth season range between 1.6 in early December, 2 in late February, and 1.8 in mid-March (Fig. 5). The cambial growth season thus corresponds to a period when LAI levels are near maximum (minimum at 80%), and LAI drops rapidly after the end of the season. No significant correlations were found between biweekly cambial growth percentages and biweekly LAI estimations.

## Discussion

Arguably the most surprising result of our analysis is the short length of the cambial growth season in *Brsp*, with a maximum length of three to four months. Low sample replication (six trees) should be taken into account when interpreting the results of our study, but the sampled trees exhibited a strong synchronicity in the onset and end of the cambial growth season, leading to conclusive results about the length of the season in *Brsp* in western Zambia. The growth season duration (83–111 days; Table 1) corresponds roughly to estimations in other dry tropical forests in southern Ecuador (2–4 months; [26]) and southeastern Ethiopia (3–4 months; [27]), but is shorter than the recorded duration in temperate (78–162 days; [54], [55]), high-elevation (88–137 days; [56], [57]), and boreal forests (75–141 days; [6], [58]), and is trumped only by the short growth season length of a high-latitude site in Finland (49–63 days in [59]). To our knowledge, cambial activity studies are rare for tropical rainforest trees [60], [61] and no cambial growth duration measurements are available for comparison.

A potential explanation for the shortness of the cambial growth season is genetic modulation to overlap with the core of the wet season (December-February). The synchronicity in the onset and end of the season between individual trees supports this hypothesis. A cambial growth start later in the wet season, when between 10 and 30% of the annual precipitation has occurred (Fig. 5), could be a defense mechanism against erratic precipitation occurrence in November. A long climate record (1904–2005) from nearby Mongu meteorological station shows that November rainfall is unreliable in this region: it was lower than 60 mm (representative of dry season conditions) in more than a quarter of the years. In comparison, no years with below-60 mm rainfall occurred for the months of December, January, and February, and only seven years for March.

TWS seasonality can provide an additional explanation for the lag between the onset of the rainy season and the onset of the cambial growth season. TWS is a function of seasonal variations in transpiration (determined by LAI, temperature, and relative humidity) and water absorption [18]. In trees on sites with low soil moisture storage, such as the Kataba Forest Reserve on deep Kalahari sand soils, the latter is largely a function of precipitation seasonality. Leaf shedding in *Brsp* occurs from July to September [33], as demonstrated on our site by a minimum LAI in mid-August, and results in an increase in TWS due to reduced transpiration. This increase in TWS allows for pre-greening behaviour in *Brsp* trees, with leaf flushing, flowering, and fruiting starting in early September [32]. The concurrence of peak vegetative and generative phenology during the hot, dry pre-rains season (September-October) is likely a life-history adaptation that enables resources such as soil nutrients (e.g., N, S), potentially released by dry season fires [62], to be pre-empted before they are leached away by rainfall [41]. Another explanation for this pre-greening behavior might be that the trees show relict behavior from a former, wetter period when the wet season started earlier in the year [63].

It is possible that the depletion of resources during this pre-greening period delays the onset of cambial growth until the tree is at full photosynthetic capacity and sufficient non-mineral nutrients are available. New *Brsp* leaves show a red coloration due to the synthesis of anthocyanins after bud burst [64]. Young, red leaves are photosynthetically active, but rates are low and less than the respiratory cost of maintenance. Both leaves and photosynthetic rates develop rapidly, reaching an asymptotic value in late September [65]. This hypothesis is supported by the correspondence of the cambial growth season with the period of high LAI (Fig. 4). Moreover, vegetative and regenerative production during the pre-greening period in September reduces TWS [23], [66], which needs to be balanced by decreasing temperatures and increasing relative air humidity in early October in addition to a sufficient amount of precipitation before secondary growth can take place.

The distinct seasonality of cambial growth explains the occurrence of annual rings in *Brsp* [36]: at the end of the cambial growth season, a band of terminal parenchyma cells is formed that demarcates the boundary between this years growth and next years. Various studies [34], [35] have shown that the width of annual growth rings in *Brsp* is related to seasonal precipitation during the core of the rainy season (December-February). This period corresponds to the period of cambial growth (late November until mid-March) as determined in our study, when up to 70% of the annual precipitation fell in the year 2001–2002. Also [67] found that cambial growth of a semideciduous Caesalpinioideae species in southeastern Brazil was restricted to the wet season. Dendrometer-based studies of cambial growth in SDTFs (e.g., [18]) have further related radial stem growth to seasonal variations in precipitation. We do not find a direct influence of precipitation on tree growth at an intra-annual (bi-weekly) resolution (Fig. 4), but an indirect effect through its influence on TWS cannot be excluded and the seasonality of cambial growth warrants the restriction of potential climatic influences on tree growth to the core of the rainy season.

A substantial rainfall event occurring more than a month after the end of the cambial growth season (Fig. 5) did not re-induce xylem formation. This finding confirms the annual nature of the growth rings in *Brsp*, the formation of which appears to be regulated by TWS rather than by individual rainfall events. A comparable lack of cambial activity initiation was found in a dry season irrigation experiment in a SDTF in western Mexico [68]. Our results suggest that growth ring anomalies, which have been reported to occur in *Brsp* [34], [35], are caused by lack of xylem growth over a portion of or the whole stem circumference, rather than sequential growth phases within one year. Growth ring anomalies in the form of partially absent rings occur more frequently in older, slow growing trees [35], [69], which explains why we found no growth ring anomalies in the relatively young trees we examined for this study.

Our results show that *Brsp* cambium only produced new xylem cells during a short period of the year, but that wound tissue was produced as a reaction to the pinning injuries throughout the entire year (Fig. 3). The ability to produce scar tissue even during cambial dormancy is possibly an adaptive trait of *Brsp* to the frequent occurrence of dry season fires in the miombo woodland [41], [70]: when low-intensity fires during the dry season partially destroy the cambium of *Brsp* trees, the injuries can be quickly closed off by wound tissue, making *Brsp* relatively tolerant of early dry season fires [71].

Our findings provide tree physiological support for the annual character of growth rings in *Brsp* and their use for paleoclimate research. *Brsp* is a widespread, dominant tree species throughout south central and eastern Africa that forms annual rings [36], [72], is drought-sensitive [34], [35], and has an estimated life-span of over 150 years [35]. The *Brsp* cambial growth period is highly synchronous between trees and occurs during the core of the rainy season (December-February), thus providing optimal potential for tree-ring cross-dating and wet season precipitation reconstruction. Furthermore, we found that the cambial growth season, and thus the season of carbon sequestration, in the seasonally dry miombo woodland is limited to only two to four months. This finding can be used to refine the carbon allocation component [73], [74] in process-based terrestrial ecosystem models [75], dynamic vegetation models [76], and coupled carbon-climate models [77]. A limited mechanistic and functional understanding of carbon allocation, and carbohydrate storage in particular [78], currently restricts the accuracy of forest ecosystem models and thus of predictions of global change impacts on the terrestrial carbon cycle [79], [80], [81]. Our study can thus provide inherent phenological constraints on carbon allocation to stem growth in the miombo woodland and can contribute to a more detailed estimation of its role in the terrestrial carbon cycle.

This study thus contributes to an improved understanding of the phenological cycle of *Brsp* and provides crucial information for the evaluation of the response of this widespread vegetation type to projected climatic changes and its role in the global carbon cycle.
